# Cerebrospinal Fluid Inflammatory Cytokine Levels in Patients With Major Psychiatric Disorders: A Multiplex Immunoassay Study

**DOI:** 10.3389/fphar.2020.594394

**Published:** 2021-02-01

**Authors:** Shinsuke Hidese, Kotaro Hattori, Daimei Sasayama, Takuya Tsumagari, Tomoko Miyakawa, Ryo Matsumura, Yuuki Yokota, Ikki Ishida, Junko Matsuo, Sumiko Yoshida, Miho Ota, Hiroshi Kunugi

**Affiliations:** ^1^ Department of Mental Disorder Research, National Institute of Neuroscience, National Center of Neurology and Psychiatry, Tokyo, Japan; ^2^ Medical Genome Center, National Center of Neurology and Psychiatry, Tokyo, Japan; ^3^ Department of Psychiatry, National Center Hospital, National Center of Neurology and Psychiatry, Tokyo, Japan; ^4^ Department of Psychiatry, Teikyo University School of Medicine, Tokyo, Japan

**Keywords:** bipolar disorder, cerebrospinal fluid, cytokine, major depressive disorder, multiplex, schizophrenia

## Abstract

**Aim:** Accumulating evidence suggests that neural inflammation plays an important role in psychiatric disorders. We aimed to identify inflammatory cytokines involved in the pathophysiology of such disorders by quantifying them in cerebrospinal fluid (CSF) samples from a large sample of patients with major psychiatric disorders and healthy controls.

**Methods:** The subjects included 94 patients with schizophrenia, 68 with bipolar disorder, 104 with major depressive disorder, and 118 healthy controls, matched for age, sex, and ethnicity (Japanese). Lumbar puncture was performed to collect these CSF samples. A multiplex immunoassay was then performed to measure CSF cytokine levels using magnetic on-bead antibody conjugation for 19 inflammatory cytokines.

**Results:** CSF interferon-β level was significantly higher in total psychiatric patients than in healthy controls (corrected *p* = 0.000029). In diagnostic group comparisons, CSF interferon-β level was significantly higher in patients with schizophrenia, or bipolar disorder (corrected *p* = 0.000047 or 0.0034) than in healthy controls.

**Conclusion:** We present novel evidence that CSF IFN-β level showed prominent statistical differences between psychiatric groups and healthy controls. This suggests IFN-β as the most important player among the 19 cytokines tested here in the inflammation-related pathophysiology of major psychiatric disorders.

## Introduction

Inflammation has been suggested to play a key role in the pathogenesis and pathophysiology of psychiatric disorders (Khandaker et al., 2017; Bauer and Teixeira, 2018), including schizophrenia (Horvath and Mirnics, 2014; Na et al., 2014; Muller et al., 2015; Khandaker and Dantzer, 2016; Muller, 2018), bipolar disorder (BD) (Rosenblat and McIntyre, 2015; Walther et al., 2017), and major depressive disorder (MDD) (Sperner-Unterweger et al., 2014; Kunugi et al., 2015; Adzic et al., 2018; Misiak et al., 2018). In the pathophysiology of these disorders, a bidirectional interplay between the brain and the immune system has been suggested (Dantzer, 2018).

Cerebrospinal fluid (CSF) is the optimal biomaterial to examine the molecular status of the central nervous system (CNS) (Veening and Barendregt, 2010; Sakka et al., 2011). Previous meta-analyses have reported altered CSF cytokine levels (e.g., interleukin [IL]-1β, IL-6, and IL-8) in patients with schizophrenia, BD, or MDD (Orlovska-Waast et al., 2018; Wang and Miller, 2018). Our research group has also reported elevations in inflammation-related molecules in the CSF of patients with schizophrenia, BD, or MDD using the enzyme-linked immunosorbent assays (Sasayama et al., 2013; Hattori et al., 2015; Ishii et al., 2018). However, there are many other candidate molecules related to inflammation.

Multiplex immunoassays (Bio-Plex and V-plex®) have been validated for the simultaneous measurement of multiple cytokine levels using blood samples from patients with MDD and healthy control subjects (Belzeaux et al., 2017). The bead-based Luminex® (Bio-Rad Laboratories, Inc.) platform has been used for CSF sample to measure 8 cytokine levels in 43 patients with BD or MDD (Haroon et al., 2016), while the immunoassay-based protein array multiplex system has been used for CSF samples to measure 10 cytokine levels in 30 patients with BD and 30 controls (Soderlund et al., 2011). The electro-chemiluminescence-based V-plex® (MesoScale Discovery) platform has also been used for CSF samples to measure 10 cytokine levels in 23 patients with chronic schizophrenia and 37 controls (Schwieler et al., 2015), 5 cytokine levels in 11 patients with recent-onset schizophrenia and 12 controls (Coughlin et al., 2016), 2 cytokine levels in 19 patients with current depression and 67 subjects without depression among older women (Kern et al., 2014), and 11 cytokine levels in 16 patients with schizophrenia and 15 with affective disorders (Maxeiner et al., 2014).

Although prior studies have conducted multiplex immunoassays to measure CSF cytokine levels in psychiatric disorders (Soderlund et al., 2011; Kern et al., 2014; Maxeiner et al., 2014; Schwieler et al., 2015; Coughlin et al., 2016; Haroon et al., 2016), the highest number of cytokines measured was at once was 11, and sample sizes of patients and controls were not very large (at most 43 and 67, respectively). This warrants to obtain more solid evidence for the role of inflammatory cytokines in the pathophysiology of major psychiatric disorders. We aimed to accomplish simultaneous quantification of a much larger number of cytokines using the bead-based Luminex® in a much larger CSF sample from patients with schizophrenia, BD, or MDD and healthy controls. We hypothesized that a variety of inflammatory changes would be detected in the CSF of patients with psychiatric disorders.

## Materials and Methods

### Participants

This study involved 94 patients with schizophrenia (mean age: 40.5 ± 10.1 years, 56 males and 38 females), 68 with BD (mean age: 43.6 ± 12.2 years, 33 males and 35 females), 104 with MDD (mean age: 43.4 ± 11.0 years, 49 males and 55 females), and 118 healthy controls (mean age: 42.4 ± 15.3 years, 66 males and 52 females) who were matched for age, sex, and ethnicity (Japanese). The BD group included 22 patients with BD I and 46 with BD II. A total of 384 samples were not based on any power analyses since we have not had data on pre-obtained effect size. All participants were recruited at the National Center of Neurology (NCNP), via advertisements at the NCNP Hospital, on our website, and in local free magazines. Participants were screened for psychiatric disorders by qualified psychiatrists using the Japanese version of the Mini International Neuropsychiatric Interview (Sheehan et al., 1998; Otsubo et al., 2005). Consensus diagnoses were determined according to the criteria laid out in the Diagnostic and Statistical Manual of Mental Disorders, 4th edition (American Psychiatric Association, 1994), based on the information from the Mini International Neuropsychiatric Interview, additional unstructured interviews, and medical records, if available. Healthy controls had no history of contact with any psychiatric services. Participants were excluded if they had a medical history of CNS diseases, severe head injury, substance abuse, or mental retardation. After providing them with a description of the study, written informed consent was obtained from every participant. The study protocol was approved by the ethics committee of the NCNP. The study was performed in accordance with the Declaration of Helsinki (World Medical Association, 2013).

### Clinical Assessments

The Positive and Negative Syndrome Scale (PANSS) was used to evaluate symptoms in patients with schizophrenia (Kay et al., 1987), and the Young Mania Rating Scale was used to evaluate manic symptoms in patients with BD (Young et al., 1978). The 21-item version of the GRID Hamilton Depression Rating Scale was used to assess depressive symptoms in patients with BD and MDD (Williams et al., 2008). Symptoms were assessed by board-qualified psychiatrists. Daily doses of antipsychotics were converted to chlorpromazine-equivalent doses, and doses of antidepressants were converted to imipramine-equivalent doses according to a published guideline (Inada and Inagaki, 2015). Every patient’s medication status was recorded at the time of the lumbar puncture.

### Lumbar Puncture

Lumbar puncture was performed in the left lateral decubitus or a sitting position. Each participant received local anesthesia by lidocaine hydrochloride injection before the puncture. CSF was withdrawn from the L3–L4 or L4–L5 interspace using an atraumatic pencil-point needle (Universe 22 or 23G, 75 mm, Unisis Corp., Tokyo, Japan). The CSF was collected in a low protein absorption tube (PROTEOSAVE SS, 15 ml Conicaltube, Sumitomo Bakelite Co., Tokyo, Japan) and immediately transferred on ice. The CSF was centrifuged (4,000 × *g* for 10 min) at 4°C and the supernatant was dispended in 0.5-ml aliquots and stored at −80°C in a deep freezer. After a single melting and re-freeze of the sample for the preparation of the 96-well plates, multiplex immunoassays were performed.

### Multiplex Immunoassays

CSF protein levels were measured with the MAGPIX CCD imaging system (Bio-Rad Laboratories, Inc.) using magnetic on-bead antibody conjugation for 37 inflammatory cytokines (171AL001M, Bio-Plex Pro™ Human Inflammation Panel 1, 37-Plex, Bio-Rad Laboratories, Inc.) according to the manufacturer's instructions. CSF samples were diluted to 1:2, and 2 standard samples (S9 and S10) were newly created to extend the assay working range on the basis of the results of a verification assay. The assay was performed on the same day using 384 single CSF samples to secure a large number after confirming that the intra-run and inter-run coefficients of variance for the 37 proteins accounted for less than 10% of the variance in the verification assay (intra-run: one set, maximum 9.7%, quadruplicate; inter-run: 16 sets, maximum 5.7%, duplicate). A VIAFLO 96/384 system (INTEGRA Biosciences, Corp.) was used to apply samples simultaneously into the 96-well plates. To adjust the inter-assay variations between the 96-well plates, 13 randomly selected CSF diluted to 1:2, 2 standards (S4 and S10), and one blank were used as margin samples to fit measures of four plates to those of 1 standard plate that included 8 standard dilution (S4–10) and blank samples. Based on the measures of the margin samples, regression equations were calculated for the 37 proteins using 2-dimensional scatter diagrams between the standard and the other four plates for the inter-plate adjustment. Among the 37 assayed proteins, the measurements of 19 cytokines satisfied the following three criteria and were thus deemed reliable: 1) within the assay working range, 2) coefficients accounting for less than 15% median inter-run variance, and 3) strong Pearson’s correlation coefficients (r > 0.70) in the regression equations of the inter-plate adjustment. The assay data for the 18 remaining cytokines (i.e., chitinase3-like 1, gp130/soluble IL-6 receptor β, interferon[IFN]-γ, IL-2, IL-12(p70), IL-20, IL-22, IL-27(p28), IL-28A/IFN-λ1, IL-32, IL-34, IL-35, LIGHT/tumor necrosis factor superfamily (TNFSF) 14, matrix metalloproteinases-1, matrix metalloproteinases-2, osteopontin, pentraxin-3, and tumor necrosis factor-related weak inducer of apoptosis/TNFSF12) did not meet some of the criteria and were thus excluded from the following statistical analyses. CSF cytokine levels are represented as pg/ml.

### Statistical Analyses

Categorical and continuous variables were compared between the four diagnostic groups using chi-square tests and analysis of variance (ANOVA), respectively. CSF cytokine levels were compared between patient (both three diagnoses combined and each diagnosis) and control groups and between drug free and non-drug free groups using Mann–Whitney U tests, while effect sizes are shown using r. Kruskal-Wallis tests were used when comparing CSF cytokine levels across the four diagnostic groups. Correlation between CSF cytokine levels and clinical variables were assessed using Pearson’s correlation coefficients, while CSF cytokine levels were compared using unpaired (Student’s or Welch’s) -t tests between sexes. Correlations between CSF cytokine levels and symptom scores or CSF total protein level were assessed using Pearson’s partial correlation coefficient controlled for age, sex, and drug use (only for patients). The correlation matrix for CSF cytokine levels was also assessed with Pearson’s partial correlation coefficient controlled for age, sex, and drug use (only for patients). Bonferroni corrections for multiple testing were applied for 3 ANOVAs for comparison of clinical variables (i.e., age, BMI, and education level) between each diagnostic and control groups (*p* < 0.05/3 = 0.016), Mann–Whitney U tests, Kruskal-Wallis tests, and correlational analyses on measured 19 CSF cytokine levels (*p* < 0.05/19 = 0.0026). Hence, corrected *p*-values were calculated 19 x nominal *p*-values for group comparisons and correlational analyses on the CSF cytokine levels. All statistical tests were 2-tailed and *p* < 0.05 was considered significant. Statistical analyses were performed using the Statistical Package for the Social Sciences version 25.0 and 27.0 (IBM Japan, Ltd., Tokyo, Japan).

## Results

### Association Between Clinical Variables and Cerebrospinal Fluid Cytokine Levels

The clinical characteristics of the participants are shown in Table 1. There were no significant differences in the distributions of age and sex, while body mass index (BMI) and education level were significantly higher and lower, respectively, in patients with schizophrenia than in healthy controls (corrected *p* = 0.004 and 0.006). There were 11, 5, and 26 drug-free patients with schizophrenia, BD, and MDD, respectively. Correlations between CSF cytokine levels and clinical variables in patients with schizophrenia, BD, or MDD and healthy controls are shown in Supplementary Tables S1–S4, respectively. BMI was significantly and negatively correlated with CSF IL-12(p40) level in patients with schizophrenia (corrected *p* < 0.05). Age was significantly and positively correlated with CSF soluble CD163 and soluble TNF-receptor one levels, while age of onset was significantly and positively correlated with CSF soluble CD163 level in patients with MDD (corrected *p* < 0.05).

**TABLE 1:** The clinical characteristics of the participants.

	Schizophrenia (*n* = 94)			Bipolar disorder (*n* = 68)			Major depressive disorder (*n* = 104)				Control (*n* = 118)		
	Mean ± Standard deviation	Range		Mean ± Standard deviation	Range		Mean ± Standard deviation	Range			Mean ± Standard deviation	Range	
Age (years)	40.5 ± 10.1		18-65	43.6 ± 12.2		20-74	43.4 ± 11.0		18-71	42.4 ± 15.3			19-77
Sex, male (%)	56 (59.6)			33 (48.5)			49 (47.1)			66 (55.9)			
Body mass index (kg/m^2^)	24.7 ± 5.5		15.3-46.7	23.9 ± 4.8		13.9-35.6	22.3 ± 3.4		15.6-33.8	22.6 ± 3.4			15.8-32.5
Education (years)	13.6 ± 2.7		9-22	14.8 ± 2.6		9-21	15.0 ± 2.5		10-26	14.9 ± 2.6			10-23
Age of onset (years)	24.2 ± 7.5		5-46	31.5 ± 11.5		15-57	33.9 ± 11.2		13-59				
Duration of illness (years)	15.7 ± 9.6		2-47	10.4 ± 7.7		0-30	7.2 ± 7.6		0-38				
Chlorpromazine-equivalent dose (mg/day)													
Total	950.7 ± 929.9		0-2750.0	175.0 ± 303.1		0-1409.1	70.0 ± 156.7		0-823.0				
Typical	99.8 ± 387.1		0-2750.0	7.4 ± 25.9		0-150.0	6.3 ± 25.1		0-150.0				
Atypical	850.8 ± 715.7		0-3645.5	171.3 ± 306.1		0-1409.1	63.7 ± 150.3		0-803.0				
Imipramine-equivalent dose (mg/day)	21.4 ± 46.4		0-225.0	51.9 ± 99.1		0-456.3	164.0 ± 143.0		0-525.0				
Drug free, n (%)	11 (11.7)			5 (7.3)			26 (25.0)						
Positive and negative syndrome scale													
Total	61.0 ± 16.1		33-115										
Positive	14.2 ± 5.1		7-27										
Negative	16.3 ± 5.2		7-28										
General	30.5 ± 8.9		16-60										
Young mania rating scale				6.2 ± 7.5		0-33							
Hamilton depression rating scale, 21-item version				11.4 ± 7.7		0-35	11.3 ± 9.2						

Drug free was counted if psychotropic medication was not used.

### Group Comparisons of Cerebrospinal Fluid Cytokine Levels

**FIGURE 1:**
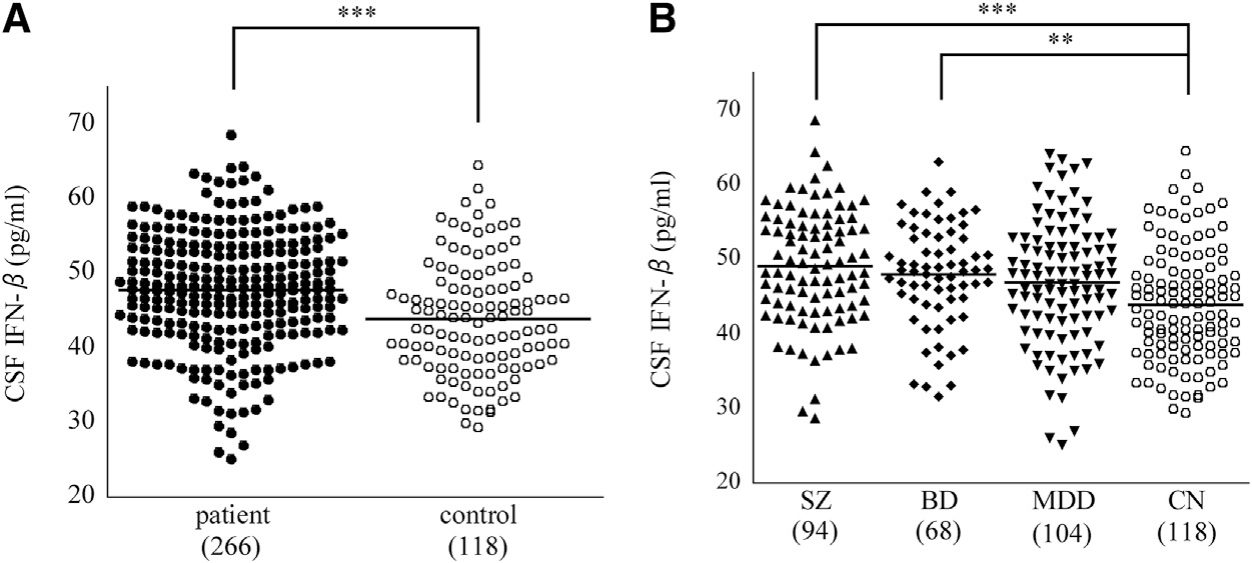
Dot plots showing that cerebrospinal fluid (CSF) cytokine interferon (IFN)-β level. CSF IFN-β level was significantly higher in the patient (3 diagnoses combined) group than in the control group **(A)**. CSF IFN-β level was significantly higher in patients with SZ, or BD than in healthy controls **(B)**. Horizontal lines in the dot plots signify mean values, while numbers in parentheses represent those of the participants. **p < 0.01, ***p < 0.001 (corrected). BD, bipolar disorder; CN, control; MDD, major depressive disorder; SZ, schizophrenia.

Comparisons of CSF cytokine levels between the patient group (3 diagnoses combined; *n* = 266) and the control group are shown in Table 2. CSF IFN-β level was significantly higher in the psychiatric patients than in the controls (corrected *p* = 0.000029, Figure 1A). Comparisons across the four diagnostic groups are shown in Table 3. CSF IFN-β level was significantly higher in patients with schizophrenia or BD than in healthy controls (corrected *p* = 0.000047 or 0.0034, respectively, Figure 1B). Comparisons of CSF cytokine levels between drug free and non-drug free patients with schizophrenia, BD, or MDD are shown in Supplementary Tables S5–S7, respectively. CSF IL-11 level was significantly lower in drug free patients than in non-drug free patients with schizophrenia (corrected *p* < 0.05).

**TABLE 2:** Comparisons of cerebrospinal fluid cytokine levels between patient (three diagnoses combined) and control groups.

	Patient (*n* = 266)		Control (*n* = 118)	Statistical comparison
	Mean ± Standard deviation		Mean ± Standard deviation	
APRIL/TNFSF13	20,589.0 ± 6,672.6	19,726.6 ± 5,419.7		U = 14710, p = 0.33, r = −0.051
BAFF/TNFSF13B	3,017.6 ± 905.4	2,811.3 ± 561.4		U = 14178.5, p = 0.13, r = −0.078
Soluble CD30/TNFSF8	347.3 ± 158.5	308.2 ± 136.0		U = 13401, p = 0.022, r = −0.117
Soluble CD163	3,514.1 ± 1,161.8	3,229.2 ± 1,151.9		U = 13474, p = 0.027, r = −0.113
IFN-α2	5.2 ± 2.6	5.0 ± 3.2		U = 14792, p = 0.37, r = −0.046
IFN-β	48.0 ± 7.8	44.0 ± 7.6		U = 10868.5, **p = 1.5.E−06**, r = −0.246
Soluble IL-6 receptor α	993.4 ± 299.3	975.9 ± 280.7		U = 15275.5, p = 0.67, r = −0.022
IL-8	23.7 ± 4.1	22.8 ± 4.5		U = 13888, p = 0.07, r = −0.092
IL-10	5.5 ± 1.4	5.6 ± 1.3		U = 15104, p = 0.56, r = −0.032
IL-11	3.5 ± 1.0	3.4 ± 1.4		U = 13753, p = 0.053, r = −0.099
IL-12 (p40)	42.2 ± 10.9	44.3 ± 9.7		U = 13980, p = 0.09, r = −0.088
IL-19	20.2 ± 2.7	20.2 ± 2.9		U = 15500.5, p = 0.85, r = −0.010
IL-26	37.7 ± 14.9	36.6 ± 17.1		U = 15052.5, p = 0.52, r = −0.033
IL-29/IFN-λ1	151.1 ± 44.5	160.0 ± 40.8		U = 13880, p = 0.07, r = −0.093
Matrix metalloproteinase-3	730.2 ± 84.1	715.8 ± 87.8		U = 14392.5, p = 0.19, r = −0.067
Osteocalcin	94.0 ± 52.5	88.6 ± 44.9		U = 14926, p = 0.44, r = −0.040
soluble TNF-receptor 1	999.1 ± 289.4	957.3 ± 243.4		U = 14902.5, p = 0.43, r = −0.041
Soluble TNF-receptor 2	365.5 ± 173.3	336.5 ± 170.5		U = 14322, p = 0.17, r = −0.070
TSLP	22.0 ± 8.2	21.2 ± 10.0		U = 14640.5, p = 0.29, r = −0.054

APRIL, A proliferation-inducing ligand; BAFF, B-cell activating factor; CD, cluster of differentiation; IFN, interferon; IL, interleukin; TNF, tumor necrosis factor; TNFSF, tumor necrosis factor superfamily; TSLP, thymic stromal lymphopoietin. A correctedly significant p-value is shown in a bold exponent (p < 0.0026), while nominally significant p-values are shown in underlined cases (p < 0.05). Values are represented as pg/ml.

**TABLE 3:** Comparisons across the four diagnostic groups.

	Schizophrenia (*n* = 94)		Bipolar disorder (*n* = 68)		Major depressive disorder (*n* = 104)		Control (*n* = 118)	Statistical comparison
	Mean ± Standard deviation	vs. control	Mean ± Standard deviation	vs. control	Mean ± Standard deviation	vs. control	Mean ± Standard deviation	
APRIL/TNFSF13	20,072.0 ± 6,131.0	U = 5341, p = 0.64, r = −0.032	20,660.2 ± 7,234.7	U =3847, p = 0.64, r = −0.035	21,009.7 ± 6,792.5	U = 5522, p = 0.20, r = −0.087	19,726.6 ± 5,419.7	H = 1.65, p = 0.65
BAFF/TNFSF13B	29,25.7 ± 892.0	U = 5350, p = 0.66, r = −0.031	3,076.3 ± 835.7	U = 3442.5, p = 0.11, r = −0.119	3,062.5 ± 961.5	U = 5386, p = 0.12, r = −0.106	2,811.3 ± 561.4	H = 3.85, p = 0.28
Soluble CD30/TNFSF8	367.2 ± 175.5	U = 4590, p = 0.031, r = −0.148	344.3 ± 154.4	U = 3432, p = 0.10, r = −0.121	331.3 ± 143.7	U = 5379, p = 0.11, r = −0.107	308.2 ± 136.0	H = 6.18, p = 0.10
Soluble CD163	3,487.8 ± 1,154.3	U = 4841.5, p = 0.11, r = -0.110	3,604.1 ± 1,236.6	U = 3324, p = 0.052, r = −0.143	3,479.0 ± 1126.1	U = 5308.5, p = 0.08, r = −0.117	3,229.2 ± 1,151.9	H = 5.18, p = 0.16
IFN-α2	5.6 ± 2.5	U = 4842.5, p = 0.11, r = -0.109	5.1 ± 2.7	U = 3946.5, p = 0.85, r = −0.014	5.0 ± 2.5	U = 6003, p = 0.78, r = −0.019	5.0 ± 3.2	H = 3.18, p = 0.37
IFN-β	49.1 ± 7.7	U = 3452.5, **p = 2.4.E−06**, r = −0.325	48.0 ± 6.9	U = 2689.5, **p = 1.8.E−04**, r = −0.275	46.9 ± 8.3	U = 4726.5, p = 0.0032, r = −0.199	44.0 ± 7.6	H = 26.57, **p = 7.3.E−06**
Soluble IL-6 receptor α	965.1 ± 265.3	U = 5453.5, p = 0.84, r = −0.015	947.5 ± 261.6	U = 3828.5, p = 0.60, r = −0.039	1,048.9 ± 342.2	U = 5441.5, p = 0.15, r = −0.098	975.9 ± 280.7	H = 4.11, p = 0.25
IL-8	24.3 ± 3.8	U = 4293.5, p = 0.0046, r = −0.194	23.5 ± 4.2	U = 3714.5, p = 0.40, r = −0.062	23.4 ± 4.4	U = 5880, p = 0.59, r = −0.036	22.8 ± 4.5	H = 8.36, p = 0.039
IL-10	5.4 ± 1.5	U = 5374.5, p = 0.70, r = −0.024	5.4 ± 1.6	U = 3805.5, p = 0.56, r = −0.043	5.5 ± 1.3	U = 5924, p = 0.66, r = 0.030	5.6 ± 1.3	H = 0.41, p = 0.94
IL-11	3.4 ± 0.8	U = 4692, p = 0.054, r = -0.133	3.4 ± 0.9	U = 3697.5, p = 0.37, r = −0.066	3.5 ± 1.2	U = 5363.5, p = 0.11, r = −0.109	3.4 ± 1.4	H = 4.62, p = 0.20
IL-12 (p40)	41.0 ± 11.7	U = 4699.5, p = 0.056, r = -0.132	42.1 ± 11.0	U = 3493, p = 0.14, r = −0.108	43.3 ± 10.1	U = 5787.5, p = 0.47, r = −0.050	44.3 ± 9.7	H = 4.73, p = 0.19
IL-19	20.8 ± 2.6	U = 4866, p = 0.13, r = −0.106	20.2 ± 2.9	U = 3936.5, p = 0.83, r = −0.016	19.7 ± 2.6	U = 5574, p = 0.24, r = −0.079	20.2 ± 2.9	H = 7.62, p = 0.055
IL-26	39.8 ± 13.9	U = 4971.5, p = 0.20, r = −0.089	38.5 ± 15.2	U = 3698, p = 0.38, r = −0.066	35.3 ± 15.4	U = 5889, p = 0.61, r = −0.035	36.6 ± 17.1	H = 4.05, p = 0.26
IL-29/IFN-λ1	149.3 ± 47.7	U = 4759, p = 0.08, r = −0.122	147.0 ± 45.2	U = 3288.5, p = 0.041, r = −0.151	155.3 ± 41.0	U = 5832.5, p = 0.53, r = −0.043	160.0 ± 40.8	H = 5.64, p = 0.13
Matrix metalloproteinase-3	731.4 ± 81.7	U = 5138, p = 0.36, r = −0.064	724.4 ± 79.5	U = 3764.5, p = 0.48, r = −0.052	732.9 ± 89.5	U = 5490, p = 0.18, r = −0.091	715.8 ± 87.8	H = 2.00, p = 0.57
Osteocalcin	96.7 ± 54.7	U = 5148, p = 0.37, r = −0.062	88.6 ± 54.7	U = 3931, p = 0.82, r = −0.017	95.0 ± 49.1	U = 5685, p = 0.35, r = −0.064	88.6 ± 44.9	H = 1.94, p = 0.59
Soluble TNF-receptor 1	971.2 ± 287.6	U = 5418, p = 0.77, r = −0.020	997.5 ± 279.8	U = 3858.5, p = 0.66, r = −0.032	1,025.4 ± 297.4	U = 5370, p = 0.11, r = −0.108	957.3 ± 243.4	H = 3.65, p = 0.30
Soluble TNF-receptor 2	347.6 ± 163.0	U = 5338.5, p = 0.64, r = −0.033	374.6 ± 179.5	U = 3548, p = 0.19, r = −0.097	375.8 ± 178.5	U = 5435.5, p = 0.14, r = −0.099	336.5 ± 170.5	H = 3.10, p = 0.38
TSLP	22.9 ± 7.5	U = 4871, p = 0.13, r = −0.105	21.9 ± 10.1	U = 3885, p = 0.72, r = −0.027	21.2 ± 7.2	U = 5884.5, p = 0.60, r = −0.036	21.2 ± 10.0	H = 2.36, p = 0.50

APRIL, A proliferation-inducing ligand; BAFF, B-cell activating factor; CD, cluster of differentiation; IFN, interferon; IL, interleukin;TNF; tumor necrosis factor; TNFSF, tumor necrosis factor superfamily; TSLP, thymic stromal lymphopoietin Correctedly significant p-values are shown in bold exponents (p < 0.0026), while nominally significant p-values are shown in underlined cases (p < 0.05). Values are represented as pg/ml.

### Correlational Analyses of Cerebrospinal Fluid Cytokine Levels

Correlations between CSF cytokine levels and symptom scores are shown in Supplementary Table S8. Significantly negative correlations between IL-11, IL-29/IFN-λ1, and thymic stromal lymphopoietin (TSLP) levels and PANSS scores were found in patients with schizophrenia (corrected *p* < 0.05), while no significant correlations were found in patients with BD or MDD. Correlations between CSF cytokine and total protein levels are shown in Supplementary Table 9S. There were no significant correlations in patients with schizophrenia or BD or in healthy controls, while CSF osteocalcin level was significantly and positively corelated with CSF total protein level in patients with MDD (corrected *p* < 0.05). Correlation matrices for CSF cytokine levels are shown in Supplementary Tables 10S–S13. As expected, many CSF cytokine levels showed significantly positive correlation with each other in patients with schizophrenia, those with BD, those with MDD, and healthy controls (corrected *p* < 0.05).

## Discussion

To our knowledge, this is the largest multiplex immunoassay study in terms of both the number of cytokines measured (*n* = 19) and the patient (schizophrenia: *n* = 94, BD: *n* = 68, MDD: *n* = 104) and controls (*n* = 118) sample size. Among the 19 reliably measured cytokines, CSF IFN-β level was higher in psychiatric patients than in healthy controls. Even when comparing across all four diagnostic groups, we found that CSF IFN-β level was higher in patients with schizophrenia and in those with BD, in this order, than in healthy controls. Notably, CSF IFN-α2 and IFN-λ1 levels were not significantly altered. These results suggest that CSF IFN-β could be a useful biomarker for psychiatric disorders.

When all four diagnostic groups were compared with the controls, the elevation in IFN-β level reached the statistical significance even after corrections for multiple testing, suggesting that elevation of IFN-β could be involved in the pathophysiology of schizophrenia. Some earlier studies suggested that IFN-γ, rather than IFN-β, could be a trait maker of schizophrenia; the source, however, included peripheral IFN-γ (Na et al., 2014; Steiner et al., 2014). A meta-analysis (Miller et al., 2011) and a review (Mohammadi et al., 2018) reported CSF cytokine levels in patients with schizophrenia, but did not include data on CSF IFN-β level. Regarding other cytokines, CSF IL-6 level was found to be elevated (Coughlin et al., 2016), while CSF IL-8 level was unaltered in patients with schizophrenia compared to controls (Schwieler et al., 2015) in the V-plex® assays, which, since IL-6 is synonymous to IFN-β2, is consistent with the findings of our study.

CSF IFN-β level was higher in patients with BD than in healthy controls. Although IFN has been suggested as an inflammatory cytokine in patients with BD (Rosenblat and McIntyre, 2015), IFN-β was not identified as a significantly elevated cytokine in a recent systematic review (Knorr et al., 2018). The present study indicates that CSF IFN-β could be involved in the pathophysiology of BD as well. Regarding other cytokines, increased CSF IL-1β and decreased CSF IL-6 levels in patients with BD compared with healthy volunteers were found in a multiplex assay (Soderlund et al., 2011). While IL-1β was not assayed in this study, our finding on IL-6 (i.e., IFN-β2) are in contrast to this earlier study. This inconsistency may be, due at least in part, to the fact that the study of Soderlund et al. (2011) examined euthymic patients with BD. To draw any firm conclusions, further studies in a large sample with the stratification by patients’ state will be necessary.

Although statistical significance could not be obtained (corrected *p* = 0.061), CSF IFN-β level was tended to be higher in patients with MDD than in healthy controls. In line with this finding, CSF IL-6 (i.e., IFN-β2) level was increased in a geriatric depression group compared with a non-depression group in an earlier V-plex® assay (Kern et al., 2014). Remarkably, exogenous IFN-α and IFN-β used for hepatitis C treatment have been reported to induce depressive states as a side effect (Loftis and Hauser, 2004; Machado et al., 2017); this is partially in accordance with our data, since CSF IFN-α2 and IFN-λ1 levels showed no significant differences between our patients and controls. Two multiplex immunoassays (Luminex® and V-plex®) reported CSF cytokine levels (Maxeiner et al., 2014; Haroon et al., 2016) in patients with affective/mood disorder; however, levels for healthy controls were not reported.

We found, for the first time, that soluble CD163 was elevated in the total psychiatric group, compared with the controls, although this finding was statistically nominal. A previous clinical study reported that CD163-positive perivascular macrophages, which is a feasible origin of soluble CD163, were abundantly detected in the brain of patients with schizophrenia with high inflammation (Cai et al., 2018). Relatedly, a macrophage-derived product neopterin (Oxenkrug, 2011) level were reported to increase in the CSF of patients with psychiatric disorders (Bechter et al., 2010; Kuehne et al., 2013; Ghisoni and Latini, 2015). Our results of increases in CD163 thus imply activation of monocytes-macrophages and may provide additional support for the involvement of inflammation in psychiatric disorders.

Negative correlations were observed between CSF IL-11, IL-29/IFN-λ1, and TSLP levels and PANSS scores in patients with schizophrenia. Unexpectedly, these results suggest that brain inflammation is not positively associated with symptom severity in patients with psychiatric disorders, which is inconsistent with previous reports including animal models (Loftis et al., 2010; Na et al., 2014; Muller et al., 2015; Rosenblat and McIntyre, 2015; Adzic et al., 2018; Misiak et al., 2018; Muller, 2018). Considering the fact that the correlations were sporadic and that significant cytokines that were identified as significant in group comparisons (i.e., IFN-β) were not included, the effects of CSF cytokines on symptom severity in schizophrenia are considerably limited. Taken together with the results of our group comparisons, these findings suggest that IFN-β is related with the pathology, but not with symptom severity, in patients with psychiatric disorders.

No significant correlations were observed between CSF cytokine and total protein levels in patients and controls, despite CSF osteocalcin level showing a positive correlation only in patients with MDD. Although direct relationship between CSF and plasma cytokine levels could not presented in this study, blood-brain barrier dysfunction and CSF total protein elevation have been reported in psychiatric disorders (Orlovska-Waast et al., 2018; Pollak et al., 2018). Since plasma cytokine levels are generally equal or higher than CSF cytokine levels (Maxeiner et al., 2014; Coughlin et al., 2016; Haroon et al., 2016), the absence of a correlation between CSF cytokine and total protein levels may suggest that the origin of most of cytokines measured in this study was central rather than peripheral. This supports a role of central inflammation in the pathology of psychiatric disorders (Na et al., 2014; Anderson and Maes, 2017).

There are following limitations. First, the majority of patients (schizophrenia: 88.3%, BD: 92.7%, MDD: 75.0%) had taken some form of psychotropic medication, although the effects on CSF cytokines were confirmed to be small in statistical comparisons. Second, 19 (51.4%) among the 37 cytokines could only be measured using a Bio-Plex assay kit. Similarly, prior studies have reported that a variety of targeted cytokines cannot be measured with V-plex® (Schwieler et al., 2015; Coughlin et al., 2016) or multiplex (Soderlund et al., 2011) assays. This implies that multiplex immunoassays are not flawless when it comes to measuring, especially if they have been designed for measurements of plasma or other tissues that contain higher levels of target molecules than the CSF. Third, significant biomarkers were rarely reported among limited cytokines measured in this multiplex immunoassay, suggesting that relatively lower number of immunocompetent cells may be involved in the pathology of psychiatric disorders. Otherwise, any prominent biomarkers may exist among cytokines which have not been included in the measurement of this study. Fourth, CSF may not be the best biological sample for seeking cytokine abnormalities in patients with psychiatric disorders. Peripheral metabolic and microbiome alternations (Aizawa et al., 2016; Rosenblat and McIntyre, 2017; Hidese et al., 2018; Horne and Foster, 2018; Kraeuter et al., 2020) may rather be more sensitive than central immunological disturbances to elucidate inflammatory changes in the pathogenesis of psychiatric disorders. Fifth, since general laboratory data about inflammatory status (e.g., CSF or plasma C-reactive protein levels) were not included, this study could not address systemic inflammation in patient groups. Sixth, this study revealed that IFN-β was significantly increased in patients with psychiatric disorders; however, dot plots of patient (both all and each diagnostic) and control groups are widely overlapped. Therefore, IFN-β will not still be used as a biomarker to distinguish ‘psychiatric disease’ from ‘normal population’. Finally, the cross-sectional nature of this study does not allow us to clearly link the etiology of psychiatric disorders to CSF inflammatory cytokines. Further studies are warranted to address the pathogenesis of brain inflammation in patients with psychiatric disorders.

In conclusion, CSF IFN-β level showed most prominent increases in psychiatric groups compared with healthy controls. Our data suggest that IFN-β could be a useful biomarker in the inflammation-related pathophysiology of major psychiatric disorders.

## Data Availability

The original contributions presented in the study are included in the article/Supplementary Material, further inquiries can be directed to the corresponding author.

## Abbreviations:

ANOVA, analysis of variance; BD, bipolar disorder; BMI, body mass index; CD, cluster of differentiation; CNS, central nervous system; CSF, cerebrospinal fluid; IFN, interferon; IL, interleukin; MDD, major depressive disorder; NCNP, National Center of Neurology; PANSS, Positive and Negative Syndrome Scale; TNFSF, tumor necrosis factor superfamily; TSLP, thymic stromal lymphopoietin.
